# Characterization of Arboblend V2 Nature Textured Surfaces Obtained by Injection Molding

**DOI:** 10.3390/polym15020406

**Published:** 2023-01-12

**Authors:** Simona-Nicoleta Mazurchevici, Oktawian Bialas, Teodor Daniel Mindru, Marcin Adamiak, Dumitru Nedelcu

**Affiliations:** 1 Machine Manufacturing Technology Department, Faculty of Machine Manufacturing and Industrial Management,“Gheorghe Asachi” Technical University of Iasi, 700050 Iasi, Romania; 2 Silesian University of Technology, 44-100 Gliwice, Poland; 3 Technical Sciences Academy of Romania, 010413 Bucharest, Romania

**Keywords:** Arboblend V2 nature, surface texturing, WCA, microindentation, SEM

## Abstract

Surface texturing is an engineering technology used in order to improve the surface characteristic of plastic parts obtained by injection molding. Applying this process not only changes the part surface properties, but also its topography. The novel functionalities of plastic products become useful when other materials make contact with the textured surface. Of course, these characteristics may vary depending on the laser positioning, dimensions, and geometry of the texture. The present paper presents the surface characteristics obtained after the laser texturing of the Arboblend V2 Nature biodegradable polymer. Three distinct geometries were studied: hexagonal, square, and triangular, and different behaviors of them were highlighted during surface free energy (SFE) and contact angle (WCA) measurements: a hydrophobic character for square and hexagonal geometry with distilled water as the measure liquid, and a hydrophilic character with diiodomethane as the measure liquid; for triangle geometry, the contact angle measurements were impossible to extract because the drop turns into a flat puddle. Additionally, the friction coefficient varied depending on the geometry texture, with the lowest value being recorded by the sample with hexagonal geometry. The micro-indentation tests highlighted increased surface micro-hardness compared to the basic material. The possibility of use in the practice of textured surfaces is viable; thus, based on the obtained results, there is even the possibility to replace non-biodegradable polymers from different sectors of activity.

## 1. Introduction

The laser surface texturing (LST) process uses laser beams focused on the surfaces to be processed, with beam diameter varying from a few to hundreds, and even thousands, of microns, with the process taking place in the air atmosphere [1]. However, the influence of the working atmosphere should not be neglected because the chemistry and wetting of the surfaces can be influenced. An example was studied by Pfleging who showed that by replacing He with an O_2_ atmosphere, there is an important increase in the wettability ofpoly(methyl methacrylate) (PMMA) [2].

The wettability of materials can be controlled by the topography (roughness) of the surfaces. Thus, Wenzel [3] demonstrated the fact that the hydrophobic character of a surface is accentuated when the roughness of the surface increases, due to the fact that the liquid penetrates the cavities of the texture. Other researchers, such as Cassie and Baxter [4] started from the assumption that the liquid does not penetrate the textures, which means that air voids tend to increase the hydrophobic character independently of its wettability. Surfaces with high roughness are easier to analyze using this model, and those with low roughness should be analyzed according to the Wenzel model [5].

Not all biodegradable polymer materials are used in industrial practice. Thus, the most used are as follows:

− Poly(Etheretherketone) (PEEK), with a semi-crystalline structure, has very good mechanical properties and high chemical resistance, being recommended for use in medical applications. This material is biologically inert precisely because of its high chemical stability and low wettability. The functionalization of textured PEEK surfaces using LST was achieved with remarkable results using laser wavelengths ranging from UV (355 nm) to mid-infrared (MIR) (10.6 μm). In the case of using laser radiation of 1064 nm, a burning of the surface occurred, and by using the laser radiation of 532 nm, the material was removed. Using this laser wavelength, grooves with an average width of 100 μm were machined. The 355 nm laser radiation produced only slight surface melting [6,7,8]. The authors of [9] highlight that improving the PEEK bioactivity was based on two main strategies: surface modification (including the surface chemical and physical treatment and surface coating) and composite preparation based on the idea of keeping the mechanical properties excellent by impregnating bioactive materials into the material substrate.

As stated in another paper [10], Young’s modulus and elastic recovery increase using the plasma immersion ion implantation (PIII) of PEEK surfaces from 4.4 to 5.2 MPa and from 0.49 to 0.68 MPa, respectively. The mechanical properties vary continuously with depth, forming a graded layer where the mechanical properties returned to untreated values deep within the layer.

− The chemical surface modification of polyethylene terephthalate (PET) was performed by the authors of [11] using a KrF 248 nm excimer laser with high and low fluency (above and below the ablation threshold). Thus, they obtained the roughness size in the micron range, and the surface showed signs of global melting. Normally, the modification of the surface by high fluency led to the deposition of some yellow to black materials on the treated surface, and the modification by low fluency led to the appearance of oxidation without detecting any ablation. The SEM, surface profilometer, and UV parameter effect (wavelength and pulse energy) were used in order to study the morphology and surface of micro-holes [12].
− Polypropylene (PP) has good thermal stability and mechanical properties [13], but the surface energy is low, limiting its use in various medical applications. There is some research on increasing the surface energy by coatings, plasma treatment, and injection molding, but without obtaining remarkable results in surface modification [1]. In [14], the authors obtained good results regarding the texturing of PP surfaces with lasers having a pulse duration of fewer than 100 ns. Laser wavelengths of 1064, 532, and 355 nm were used, and a black smoke layer was used in order to increase the absorption capacity. After applying the LST process, the final roughness obtained was greater than 1 µm, and was considered the minimum value for improving the adhesion properties of the PP surfaces.
− Polycarbonate (PC) has good mechanical properties and biostability. The functionality of textured PC surfaces using LST was achieved from UV (λ = 248 nm) to NIR (λ = 1064 nm) [1]. A decrease in the surface oxygen content led to an increase in the hydrophobicity of PC surfaces if is used an extreme ultraviolet laser-plasma source, as shown through XPS analysis [15]. An increase in roughness was observed when using 1064 nm and 355 nm laser radiation, but wetting only increased when using 1064 nm laser radiation [16].
− Polytetrafluoroethylene (PTFE) was modified by a UV laser [17] in a nitrogen-rich environment. Controlled textured models with high precision were obtained.
− Nylon 6.6 was textured using a CO_2_ laser in order to increase biocompatibility [18]. Surface modification affected the cell viability of nylon, and cell growth was enhanced.
− Polyimide (PI) was textured using different laser wavelengths (λ = 1.064, 532, 355, and 266 nm) to avoid biofilm formation on existing medical devices [19].
− Poly(methyl methacrylate) (PMMA) experiments were performed using a Ti: Sapphire fs laser source (λ = 800 nm and a pulse duration of 150 fs) to evaluate wettability changes [20], recording an increase in the WCA. The method was also used to fabricate microchannels with controlled dimensions and roughness for microfluidic applications. In his research, Pfleging [2] studied the influence of the processing atmosphere on wettability during UV laser treatment (λ = 193 nm). He found a significant decrease in WCA in the case of using O2 as processing gas, due to surface oxidation. Kallepalli DLN et al. [21] obtained several microstructures (buried gratings, surface gratings, surface micro craters, and micro channels) in bulk poly(methylmethacrylate) (PMMA) and poly(dimethylsiloxane) (PDMS) using the femtosecond (fs) direct writing technique. They recommended an optimized parameter in order to achieve maximum efficient gratings for both materials. The highest diffraction efficiency (DE) recorded in the case of PDMS grating was around 10% and in the case of PMMA around 34%, obtained with an 0.65 NA (40X) objective with a single scan.

The desired outcomes of this study included the texturing of the surfaces of the parts obtained by injection into the mold from the Arboblend V2 Nature biodegradable material by using hexagonal, square, and triangular geometries and characterization of the obtained sample. The novelty of the study consists in the texturing obtained on the surface of the Arboblend V2 Nature biodegradable material and also in its improved surface properties.

## 2. Materials and Methods

### 2.1. Sample Preparation

The material of the samples used for texturing is Arboblend V2 Nature, a biopolymer patented by a team of researchers from the Fraunhofer Institute for Chemical Technology (ICT) in Pfinztal (Germany) in collaboration with the company Tecnaro GmbH, showing great potential for use in industrial applications from all fields that use renewable resources and whose content is based on biopolymers, such as polyhydroxyalkanoates (PHAs), polycaprolactone (PCL), polyester (e.g., bio-PET), starch, polylactic acid (PLA), bio-polyolefins (bio-PEs), bio-polyamides (bio-PAs), lignin, natural resins, natural waxes, natural oils, natural fatty acids, cellulose, organic additives and natural reinforcing fibers [22]. The basic properties of the Arboblend V2 Nature material are presented in Table 1 [23], and a number of authors have studied this material both from the point of view of mechanical and thermal properties and have also analyzed its structure [24,25,26,27,28,29].

**Figure 1:**
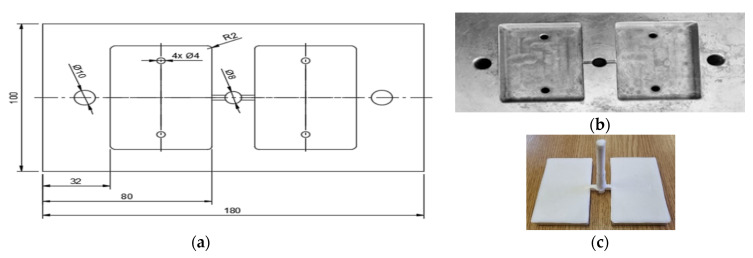
The active plate of the mold: (**a**) the dimensions of the active plate and the forming cavities; (**b**) the real image of the active plate; (**c**) parts obtained by injection molding.

The samples used for texturing were obtained by injection into the mold using the SZ-600H injection machine (Shen Zhou, Zhangjiagang, China). The dimensions of the mold active plate and the cavities are shown in Figure 1.

To obtain better quality results, homogenized sample preparation was used for each sample. Before laser micromachining, the mechanical finishing process was performed using the grinding–polishing machine, known as TERGAMIN-30 (Struers, Willich, Germany). Each sample was mechanically planed and sequentially polished with paper grain-size gradations of 500, 800, and 1200 grid/mm^2^ in time t = 4 min per each gradient, and mechanically polished by polishing wheels, with gradations of 9, 3, and 1 µm.

### 2.2. Surface Texturing

The Arboblend V2 Nature samples delivered to the laboratory were subjected to a laser texturing process using the A-355 ps micromachining laser system (Oxford Lasers, Shirley, USA). For the square and hexagonal geometries, two samples were obtained, with 2 passes and 4 passes each. The samples were conditioned in the air for 3 days, then blown with compressed air and tested according to the methodology presented below. The LST parameters were established based on initial tests on the material behavior. The process was given the following parameters presented in Table 2.

In order to measure the surface free energy (SFE) and contact angle (WCA), a test stand was used, which included OGE’s Surftronic Universal goniometer (OEG Company, Hessisch Oldendorf, Germany) and a Surface 4.5 computer to analyze the reported fall image. Drops of the measured liquids (distilled water and diiodomethane) were placed on the test sample surface, each of which had a volume of 1.5 μL. The experiments were conducted at room temperature T = 23 °C and began 20 s after the sample drops were dispensed. The Owens–Wendt–Rabel–Kaelble (OWRK) method was used with some assumptions adopted such as: the purity of the liquid, the chemical homogeneity and smoothness of the solid, and the lack of chemical reactions between the two environments (liquid and solid). In reality, not all of these conditions were met, but the OWRK method is a very good one and is used both in industry and in the academic environment to select the material.

Scanning electron microscopy (SEM) was carried out using a QUANTA 200 3D SEM-FIB electron microscope (FEI Company, Fremont, CA, USA). The following parameters were used: the secondary electrons’ acceleration voltage—30 KV; 100X-5000X magnification power; working distance—15 mm; (large field detector (LFD); tilt angle of 0°, and the pressure of the microscope chamber—60 Pa.

In order to follow the microindentation tests, a universal piece of UMT-2 (CETR-Center of Tribology Inc., Campbell, CA, USA) equipment was used with a 2 kg sensor and a maximum force of 10 N was applied. Additionally, a diamond Rockwell indenter with a radius of 200 µm was used, and the capacitive sensor measured the vertical indenter displacement. 

In order to measure the friction coefficient, the wear test was performed using the pin-on-plate method. The test was carried out using a CSM tribometer (CSM Instruments, Needham, MA, USA), equipped with an arm with a pin holder, controller, and the rotated stage.

## 3. Results and Discussion

### 3.1. Surface Free Energy (SFE) and Contact Angle (WCA) Measurements 

**Figure 2:**
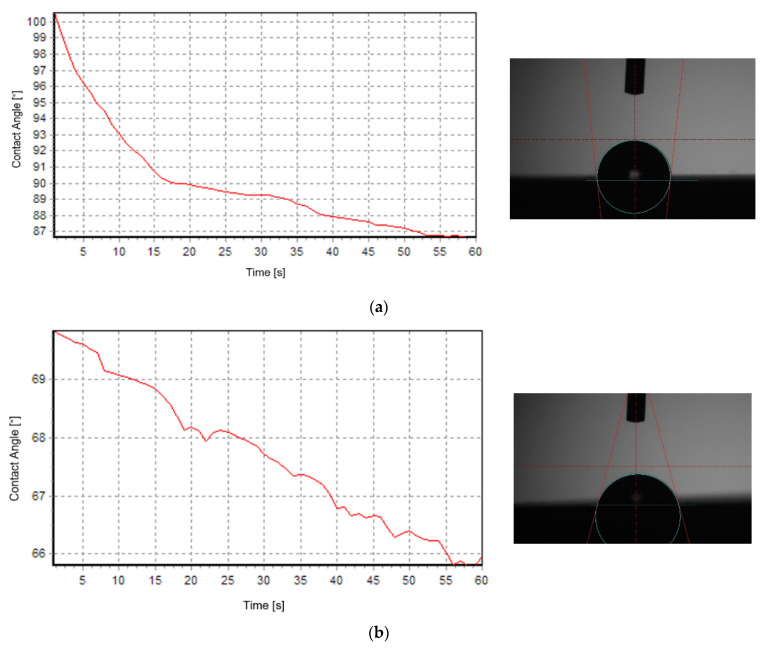
Contact angle measurements: (**a**) distilled water as the measure liquid; (**b**) diiodomethane as the measure liquid.

The values of SFE for the Owens–Wendt method and its polar and dispersion properties are given in Figure 2 and the results are presented in Table 3.

To assess the SFE, the measurements should be taken on at least two liquids. One liquid should be dominantly polar (e.g., distilled water or glycerol) and one liquid should be dispersive (e.g., diiodomethane). Calculations on the SFE are more complex, but, afterward, the total surface free energy of the solid–liquid surface should be more or less given by Relation (1):

*γ_S_* = *γ_S_^d^* + *γ_S_^p^* (1)

When using distilled water as the measure liquid, the hydrophobic character of the surface was obtained for the samples, which were double-textured, regardless of the geometry of the texture (square and hexagon geometry). Using the diiodomethane measure liquid, the surface had a hydrophilic character. A wetting angle of more than 90° pointed to the weak wettability of the surface. The highest value of WCA was obtained for the first sample group (square geometry texture), and the mean value was approximately 104°, which pointed to the hydrophobic character of the surface. Increasing the number of laser transitions (four passes) reduced the WCA, potentially caused by the decrease in the specific surface area because of the increased depth of the groves.

For the samples textured using triangle geometry, the WCA measurements were impossible to obtain regardless of the number of laser transitions. The drop turned into a flat puddle (0°), indicating very high hydrophilicity. Additionally, for the samples characterized by the hydrophobic character of the surface, the highest value of apolar components was higher compared to polar components. 

### 3.2. Microscopic Observations

**Figure 3:**
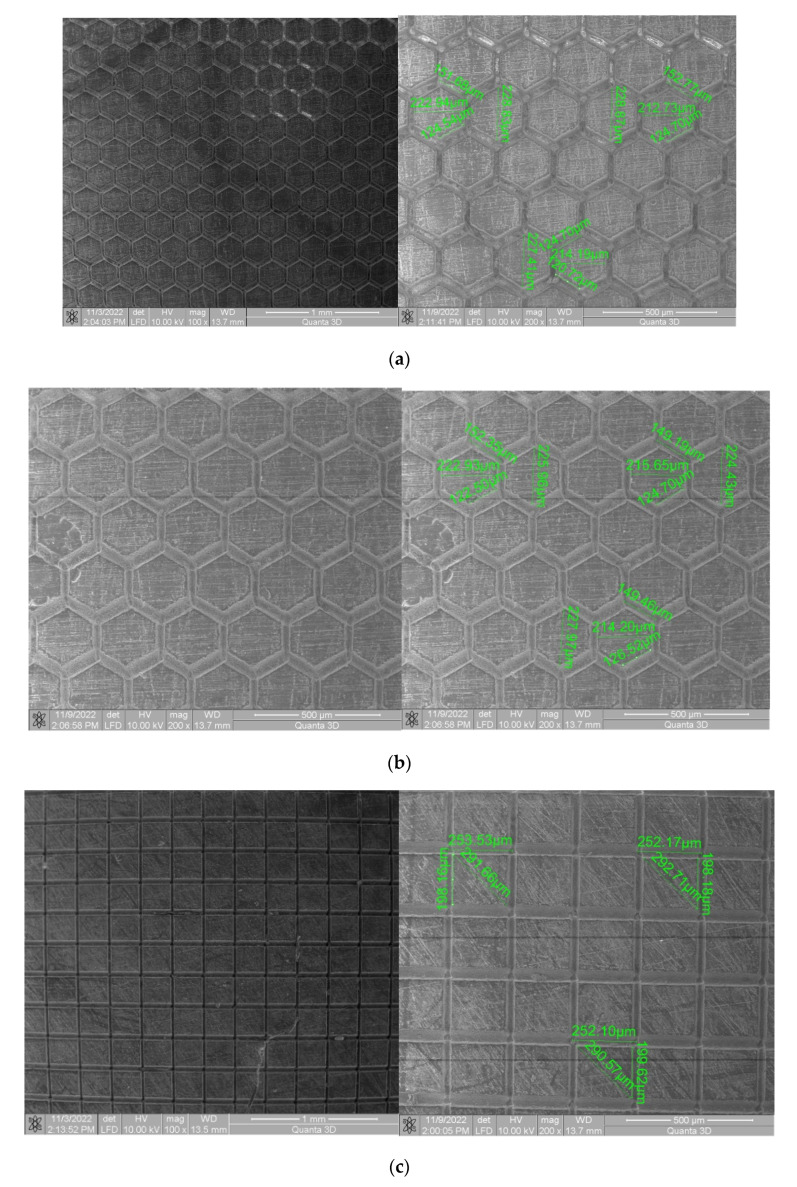

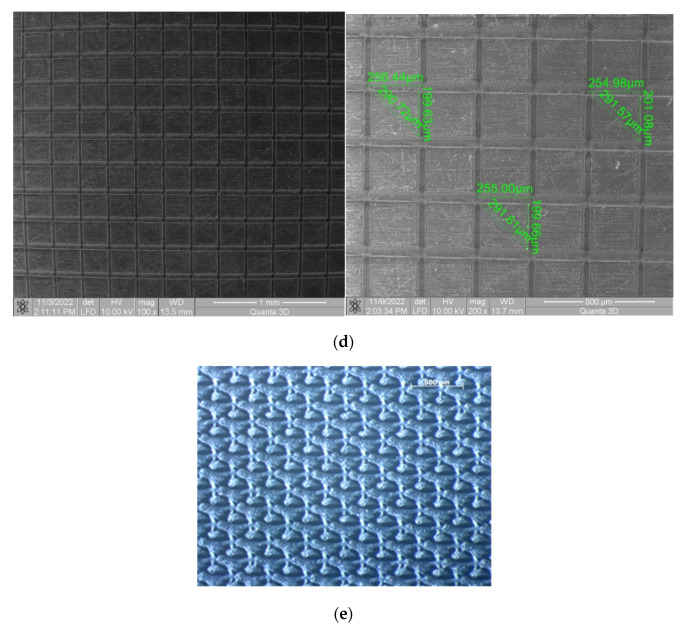
The geometries and number of passes: (**a**) hexagonal 2X; (**b**) hexagonal 4x; (**c**) square 2X; (**d**) square 4X; (**e**) triangle 4 passes.

The geometries of textures made and the number of passes are shown in Figure 3.

Regardless of the texture’s geometry, the samples with double-textured surfaces exhibited hydrophobic properties (square and hexagon geometry). The weak wettability of the surface was indicated by a wetting angle greater than 90°. The first sample group (square geometry texture) had the highest WCA value, and the mean value was close to 104°, indicating that the surface is hydrophobic. The WCA could be decreased by decreasing the specific surface area due to increasing the depth of the groves, likely accomplished by increasing the number of laser transitions. Additionally, the greatest value of apolar components was larger than polar components for the samples with hydrophobic surface characteristics. Based on this, it can be concluded that all tested samples exhibit a greater affinity to apolar groups of SFE than to polar ones.

Using SEM, the dimensions of the obtained textures were measured and an average was calculated with three measurements. Thus, in the case of the hexagonal texture with two passes, an average value of the diagonal of 228.20 µm was obtained, and in the case of the hexagonal texture with four passes, an average value of 226.12 µm was obtained. In the case of the square texture with two passes, the average value of the diagonal was 291.64 µm, and for the square texture with four passes, the average value of the diagonal was 291.3 µm. In the case of both geometries, it can be mentioned that the measured values for three different textures are comparable, which indicates very good processing precision.

In order to establish the width and depth of the grooves for each type of texture, the topography of the surfaces presented in Table 4 was found.

According to Table 4, the highest height of the grooves, 149 µm, was obtained in the case of the hexagonal texture with four passes, and the highest width of the grooves, 85.4 µm, was also obtained for the hexagonal texture but with two passes.

### 3.3. WCA Measurements 

The study was carried out using a test stand, which included a Kernco G-I Contact Angle Meter / Wettability Analyzer (El Paso, TX, USA). The experiments were conducted at room temperature T = 23 °C and began 15 s after the sample drops were dispensed. The results are presented in Table 5.

Based on the obtained results, we can conclude that the tendency for the wetting angle behavior noticed in previous wettability tests (on a different device and with a different surface preparation) was maintained, but the values differ from those obtained in the earlier tests.

### 3.4. Study of the Friction Coefficient

**Figure 4:**
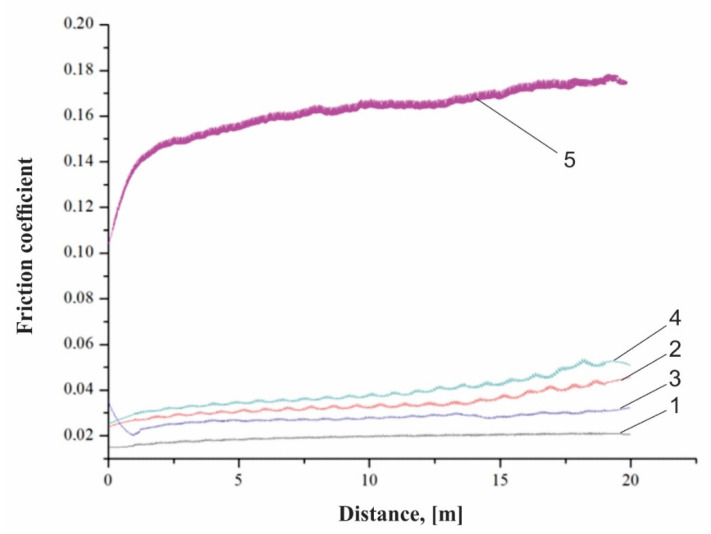
Friction coefficient diagram: 1-square 2x; 2-hexagonal 2x; 3-square 4x; 4-hexagonal 4x; 5-base material.

Three measurements were taken for each texture type, and the results were averaged. The friction coefficient values obtained are low and the results are presented in Table 6 and in Figure 4.

As can be seen from Figure 4, the square texture, regardless of the number of passes made, allowed the lowest coefficient of friction to be obtained. The hexagonal texture with two or four passes helped to obtain friction coefficients close in value. Only in the case of the square geometry, regardless of the number of passes, was there a significant reduction in the friction coefficient (four traces—0.033 and two passes—0.027) of approximately 80%, compared to the average value of the friction coefficient of the base material (0.17). In the case of the hexagonal geometry, the increase in the number of passes, from two to four, led to an increase in the friction coefficient, from 0.039 to 0.047. This may be because in the dry-friction state, it is easy to produce wear debris by grinding. Both square and hexagonal textures had the ability to accommodate the grinding products to reduce abrasive particle wear, so the friction coefficient was lower relative to the untextured surface in the case of the square geometry. However, the larger the texture area, the larger the contact pressure, causing more abrasive wear. There is no significant difference in results among the types of texture under the set of applied test conditions, while there is a significant difference compared to the untextured surface.

### 3.5. Microindentation Test

**Figure 5:**
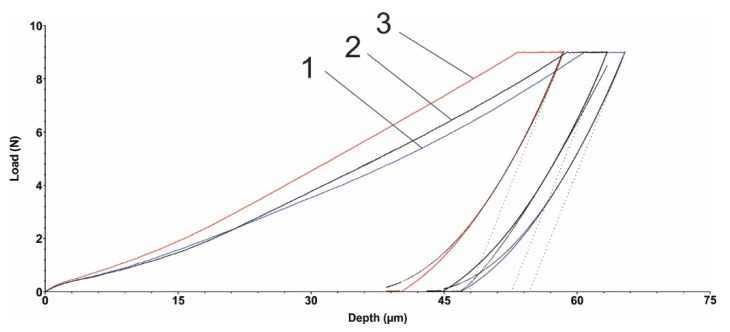
Hexagonal texture microindentation test (2 passes): 1, 2, 3—measurements.

**Figure 6:**
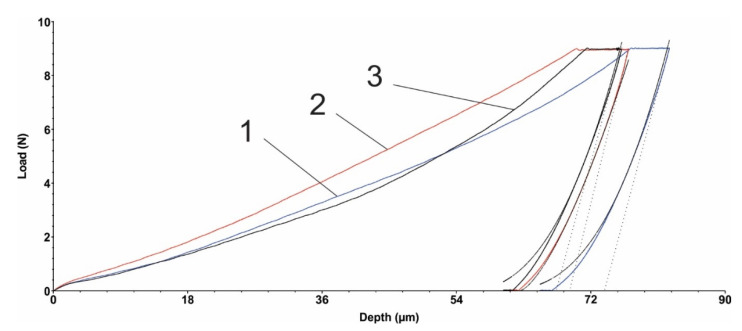
Hexagonal texture microindentation test (4 passes): 1, 2, 3—measurements.

**Figure 7:**
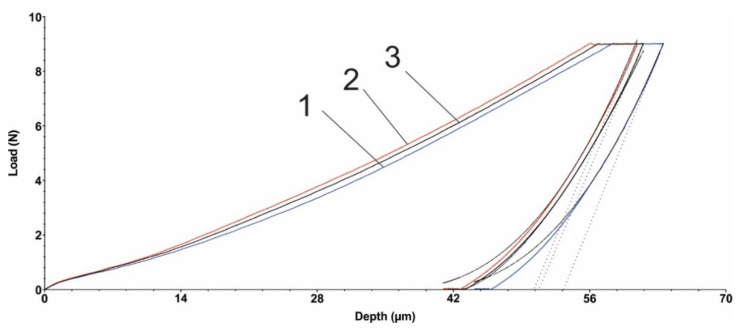
Square texture microindentation test (2 passes): 1, 2, 3—measurements.

**Figure 8:**
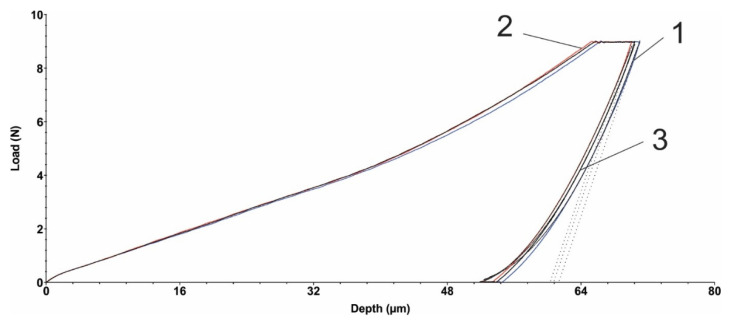
Square texture microindentation test (4 passes): 1, 2, 3—measurements.

Figure 5 and Figure 6 show the load diagrams dependent on the vertical indenter displacement for the hexagonal texture microindentation test—two passes and four passes, respectively. Figure 7 and Figure 8 show the square texture microindentation test—two passes and four passes, respectively. The software package used enabled reading the microhardness values and Young’s modulus. These values are shown in Table 7 for the hexagonal texture and Table 8 for the square texture. Both tables also include the maximum depth (µm) for each sample tested. The research was conducted on three test samples of each textured type. The studied test samples showed the following average values in the cases of hexagonal texture (Table 7) and square texture (Table 8).

As can be seen from Table 7 and Table 8, the square texturing obtained after four passes gives the material the highest deformability (with the lowest average Young’s mode value of 2.258 GPa), while the square texturing obtained after two passes gives the material the highest stiffness (with the highest average value of Young’s mode of 2.462 GPa). The average values obtained in the case of hexagonal geometry, regardless of the number of passes, are close in value. The average values of the micro-hardness are, in general, in accordance with the average obtained values of Young’s modulus, with the mention that a maximum average value of the micro-hardness was obtained both in the case of the hexagonal geometry with two passes and in the case of the square texture, also with two passes of 0.133 GPa. Comparable average values of the micro-hardness were obtained in the case of the hexagonal and square geometries obtained after four passes, with values of (0.091 ± 0.006) GPa and (0.109 ± 0.002) GPa, respectively. Regardless of the obtained geometry, in the case of texturing with two passes, the same average value of 0.133 GPa was obtained. The general statement can be made that by lowering the hardness, the coefficient of friction is being slightly increased. It can be explained by the fact that lower hardness can lead to easier and longer contact with the penetrator (counter sample being under load) and material, even though the grooves are present, the result of which is presented by an increased COF. In the case of harder surfaces, the penetrator has a lower contact area with the polymer material.

## 4. Conclusions

Laser surface texturing (LST) technology at the macro, micro, and nano levels consists of the direct treatment of polymeric biodegradable materials with a laser beam, with the possibility of modifying surface roughness and chemistry in a single step, which indicates a simultaneous modification of the surface energy without using toxic chemicals.

In the case of double-textured surfaces, both for square and hexagonal geometry, a hydrophobic character was obtained, using distilled water as the measure liquid. For the second measure liquid, diiodomethane, a hydrophilic character, was obtained for the surfaces.

For surfaces textured using triangle geometry, regardless of the number of laser transitions, the WCA measurements were impossible to measure because the drop turns into a flat puddle.

The square geometry, regardless of the number of passes made, allowed the lowest friction coefficient to be obtained, as well as the hexagonal geometry, with two or four passes, which then allowed friction coefficients close in value to be obtained.

The highest deformability of the material was recorded in the case of square geometry with four passes, with the lowest average value of Young’s modulus, and the square geometry of the texture resulting from two passes gives the material the greatest rigidity, with the highest average value of Young’s. In the case of the hexagonal geometry, the average values obtained, regardless of the number of passes, are close in value. From the point of view of the micro-hardness, the average values obtained are, in general, in accordance with the average values of Young’s modulus. It is of note that a maximum average value of the micro-hardness was obtained both in the case of the hexagonal geometry with two passes and in the case of the square geometry, also with two passes. In the case of the hexagonal and square geometry with four passes, comparable average micro-hardness values were obtained.

## Figures and Tables

**Table 1:** Comparative characteristics of Arboblend [23].

Property	Measurement Unit	V1	V2 (Standard)	V3
The flow resistance	MPa	57	49	42
The specific yield strain	%	2.4	2.3	2.4
The modulus of elasticity	MPa	2900	2700	2300
The strain at break	MPa	28	25	24
The specific strain at break	%	5.7	9.4	>10
The impact resistance	KJ/m^2^	42	59	93

**Table 2:** LST system parameters.

Parameters	
Software	Cimita laser micromachining software suite for laser, motion, and vision
Laser	Diode-pumped solid state
Cut speed	1 [mm/s]
Cut passes	2 and 4 passes
Power	48 [mW]
Pulsation frequency	400 [Hz]
Wave length	355 [nm]
Pulse width	6 [ps]

**Table 3:** SFE and WCA results.

WCA Θ [^o^]					Surface Free Energy, SFE [mJ/m^2^]		
No	Series	Passes	Measure Liquid		$\gamma_{S}^{p}$	$\gamma_{S}^{d}$	$\gamma_{S}$
			Distilled Water	Diiodomethane			
0	Initial state		45.1	34.2	17.2	29.1	33.1
1	Square	2	104.0	52.9	0.1	39.4	33.4
		4	73.3	70.8	18.9	70.4	84.9
2	Hexagonal	2	88.2	34.9	0.8	39.3	39.9
		4	69.8	77.3	28.1	6.8	34.9
3	Triangle						

**Table 4:** Confocal microscopy 3D reconstruction and roughness diagram.

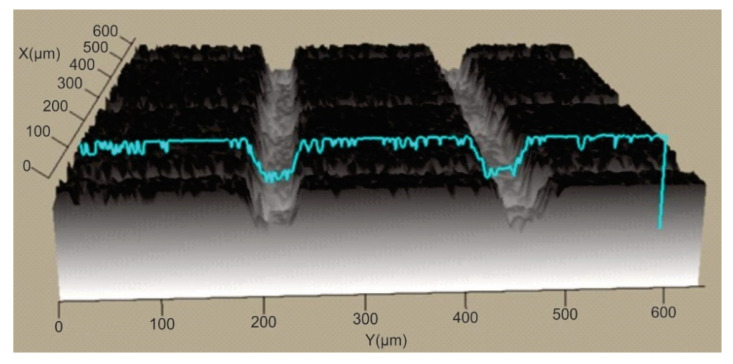			
Axis	Marker 1	Marker 2	Diference
X	183.3 µm	257.4 µm	74.1 µm
Z	187 µm	283 µm	96 µm
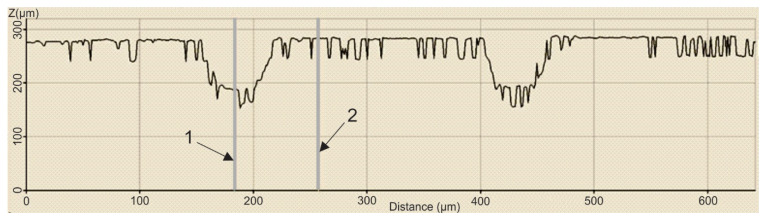			
Square 4 passes roughness profile: height of grooves, Hgroove = 96 µm; width of grooves, Wgroove = 74.1 µm.			
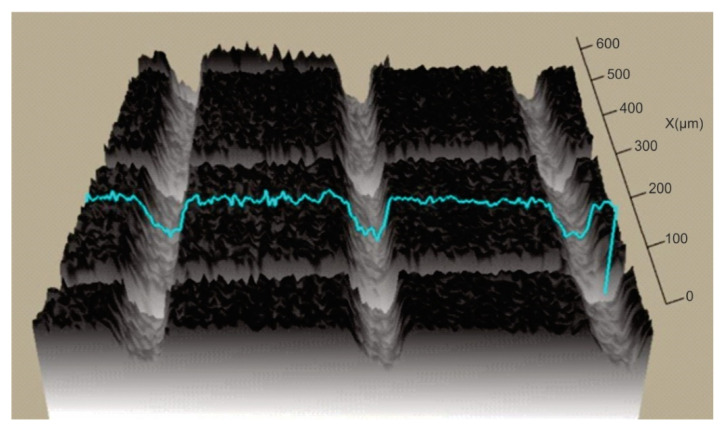			
Axis	Marker 1	Marker 2	Diference
X	224.7 µm	288.8 µm	64.0 µm
Z	98 µm	56 µm	−42 µm
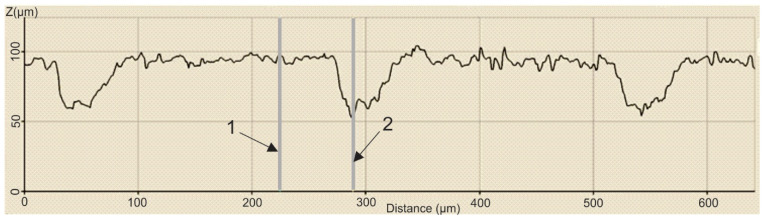			
Square 2 passes roughness profile: height of grooves Hgroove = 42 µm; width of grooves, Wgroove = 64.0 µm.			
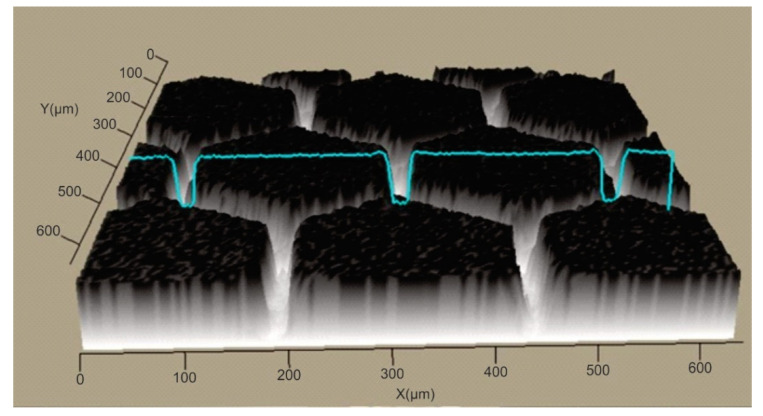			
Axis	Marker 1	Marker 2	Diference
X	316.4 µm	247.3 µm	−69.1 µm
Z	29 µm	178 µm	149 µm
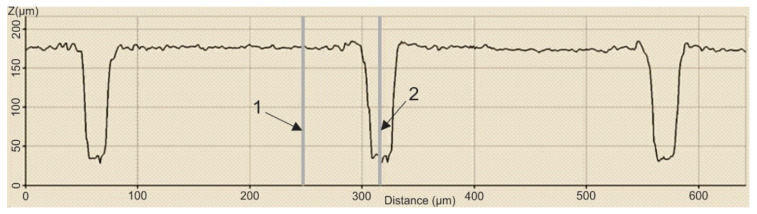			
Hexagonal 4 passes roughness profile: height of grooves, Hgroove = 149 µm; width of grooves, Wgroove = 69.1 µm.			
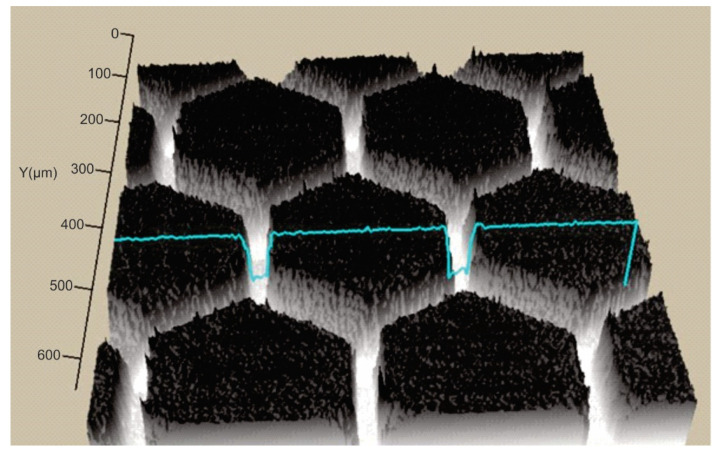			
Axis	Marker 1	Marker 2	Diference
X	170.8 µm	256.1 µm	85.4 µm
Z	33 µm	89 µm	56 µm
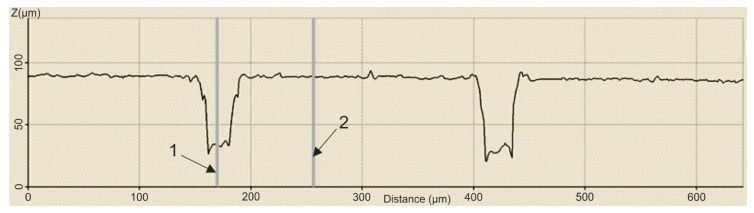			
Hexagonal 2 passes roughness profile: height of grooves, Hgroove = 56 µm; width of grooves, Wgroove = 85.4 µm.			

**Table 5:** WCA results.

Θ [^o^]				
**No**	**Series**		**Liquid: Distilled Water**	Each result is the average of 9 angle measurements approximately 15 s after droplet formation.
1	Square	2	115	
		4	106.5	
2	Hexagonal	2	86.25	
		4	56	
3	Unmodified surface	-	70.5	

**Table 6:** Coefficient of friction results.

Test No.	Unmodified Surface	Hexagonal 4 Passes	Square 4 Passes	Hexagonal 2 Passes	Square 2 Passes
1	0.17	0.05	0.03	0.044	0.02
2	0.15	0.042	0.039	0.35	0.04
3	0.19	0.49	0.031	0.39	0.022
Average	0.17	0.047	0.033	0.039	0.027

**Table 7:** Hexagonal (H) texture values.

	Test Number	Maximum Load (N)	Maximum Depth (µm)	Young’s Modulus (GPa)	Micro-Hardness (GPa)
H2x_10N Two passes	test 1	8.972	65.399	2.309	0.124
	test 2	8.969	58.469	2.461	0.147
	test 3	8.984	63.399	2.373	0.13
Average		8.975	62.422	2.381	0.133
Dev. Std.		0.008	3.567	0.076	0.012
H4x_10N Four passes	test 1	8.989	82.538	2.313	0.0844
	test 2	8.95	77.065	2.481	0.0931
	test 3	8.96	76.15	2.399	0.0956
Average		8.9663	78.584	2.397	0.091
Dev. Std.		0.020	3.454	0.084	0.006

**Table 8:** Square (S) texture values.

	Test Number	Max Load (N)	Max Depth (µm)	Young’s Modulus (GPa)	Micro-Hardness (GPa)
S2x_10N Two passes	test 1	8.998	63.57	2.4	0.129
	test 2	8.973	60.872	2.519	0.137
	test 3	8.995	61.531	2.467	0.135
Average		8.988	61.991	2.462	0.133
Dev. Std.		0.014	1.407	0.060	0.004
S4x_10N Four passes	test 1	8.959	71.058	2.24	0.108
	test 2	8.984	70.089	2.273	0.111
	test 3	8.969	70.453	2.263	0.11
Average		8.970	70.533	2.258	0.109
Dev. Std.		0.013	0.489	0.017	0.002

## Data Availability

Not applicable.
